# Assessment of Condylar Changes in Patients with Temporomandibular Joint Pain Using Digital Volumetric Tomography

**DOI:** 10.1155/2014/106059

**Published:** 2014-09-21

**Authors:** Ujwala Shivarama Shetty, Krishna N. Burde, Venkatesh G. Naikmasur, Atul P. Sattur

**Affiliations:** ^1^A J Institute of Dental Science and Hospital, Mangalore, Karnataka, India; ^2^SDM College of Dental Science and Hospital, Dharwad, Karnataka, India

## Abstract

*Objective*. To evaluate the efficiency of DVT in comparison with OPG in the assessment of bony condylar changes in patients of TMJ pain. *Methods*. 100 temporomandibular joints of 62 patients with the complaint of temporomandibular joint pain were included in the study. DVT and OPG radiographs were taken for all the 100 joints. Three observers interpreted the DVT and OPG radiograph for the bony changes separately for two times with an interval of one week. The bony changes seen in the condyle were given coding from 0 to 6. (0: Normal, 1: Erosion, 2: Flattening, 3: Osteophyte, 4: Sclerosis, 5: Resorption, and 6: other changes). Interobserver and intraobserver variability was assessed with one-way ANOVA statistics. *Z* test was used to see the significant difference between OPG and DVT. *Results*. In the present study the interexaminer reliability for OPG and DVT was 0.903 and 0.978, respectively. Intraexaminer reliability for OPG and DVT was 0.908 and 0.980, respectively. The most common condylar bony change seen in OPG and DVT was erosion followed by flattening and osteophyte. There was significant difference between OPG and DVT in detecting erosion and osteophytes. The other changes observed in our study were Ely's cyst, pointed condyle, and bifid condyle. All the bony changes are more commonly seen in females than males. *Conclusion*. DVT provides more valid and accurate information on condylar bony changes. The DVT has an added advantage of lesser radiation exposure to the patient and cost effectiveness and could be easily accessible in a dental hospital.

## 1. Introduction

Temporomandibular joint is one of the most fascinating and complex synovial systems in the body. It is the area in which the mandible articulates with the cranium [1]. The masticatory system is extremely complex, which comprises primarily of bones, muscles, ligaments, and teeth, all of which are responsible for activities like mastication, speech, and deglutition. All these movements are regulated by an intricate neurological controlling mechanism, which is important for the system to function normally and efficiently [1]. Lack of such harmony may lead to disruptive muscle behavior or structural damage to any of the components. The function of the TMJ is unique, in that the condyle both rotates within the fossa and translates anteriorly along the articular eminence. Because of the ability of the condyle to translate, the mandible can have a much higher maximal incisal opening than would be possible with rotation alone. Because of all these features, the TMJ is referred to as “ginglymodiarthrodial,” which means a combination of the terms ginglymoid (rotation) and arthrodial (translation) [2].

In UK orofacial pain accounts for approximately 10% of pain in the adult population [3]. The prevalence rates of orofacial pain in the Indian population are not available. Temporomandibular disorders (TMD) is a term given to a heterogeneous group of pathologies affecting the temporomandibular joints, the masticatory muscles, or both. It is the most commonly occurring jaw disorder, with a prevalence rate of 28% to 86% of adults and adolescents showing one or more clinical signs or symptoms [4]. The etiology of the TMD is unknown. Occlusal disharmony and psychological distress are the two hypotheses which have dominated the literature. The psychological hypothesis proposes that the disorder evolves as a consequence of psychological distress that is usually due to the individual's stressful environment. The psychological distress in turn leads to parafunctional habits (tooth clenching and grinding) that result in muscle pain [5]. There are various signs and symptoms of TMJ dysfunction. It may include one or more of the following: pain in the TMJ or ear or both, headache, muscle tenderness, joint stiffness, clicking or other joint noises, reduced range of motion, locking, and subluxation [4]. TMJ imaging may be necessary to supplement information obtained from the clinical examination. It is useful particularly when an osseous abnormality or infection is suspected; conservative treatment has failed or symptoms are worsening. Screening projection used for TMJ is panoramic projection. It provides an overall view of the teeth and jaws. It also helps in identifying odontogenic diseases and other disorders that may be the source of TMJ symptoms. Specific TMJ programs are available in some of the panoramic machines. They have the disadvantage of thick image layers and the oblique, distorted view of the joint, which severely limits image quality. Hence there is a need for advanced radiographic imaging for TMJ. The main aim for TMJ imaging includes evaluation of the integrity of the structures when disease is suspected, determination of the extent of disease and its progression, and finally evaluation of the effects of treatment [6]. Recent imaging technology for TMJ is digital volumetric tomography (DVT). It was developed for angiography in 1982 and subsequently applied to maxillofacial imaging. It uses a divergent or “cone”-shaped source of ionizing radiation and a two dimensional area detector fixed on a rotating gantry to acquire multiple sequential projection images in one complete scan around the area of interest. It is only since late 1990s that it has become possible to produce clinical systems that are both inexpensive and small enough to be used in the dental office [7]. This technology has been given several names including dental volumetric tomography, cone beam volumetric tomography, cone beam computed tomography, dental computed tomography, cone beam imaging [5], and CB3D [8]. DVT provides high definition three-dimensional digital data on precise anatomical information of all oral and maxillofacial structures at reduced cost and less radiation to patient, in comparison to traditional imaging systems, which are limited by distortion, magnification changes, restricted clarity, lack of accuracy in measurements, and not allowing for 3D reconstruction. TMJ imaging poses a challenge because the bony components are small and superimpositions from the base of the skull often result in a lack of clear delineation of the joint. Different imaging modalities have been used for TMJ but they have disadvantages such as superimpositions, high radiation dose, and long scanning time present severe limitation. These disadvantages have led to an increase in popularity of the use of DVT for TMJ imaging [9].

## 2. Materials and Methods

62 patients who visited the Department of Oral Medicine and Radiology, SDM College of Dental Sciences and Hospital, Dharwad, with chief complaint of temporomandibular joint pain were selected for the study. Their age ranged from 15 to 72 years. A total of 100 joints were assessed in these patients. Approval from the ethical board has been obtained for this study.

Inclusion criteria are as follows:

1. patients willing to participate in the study;
2. patients with a complaint of chronic temporomandibular joint pain.

Exclusion criteria are as follows:

1. systemic, rheumatic, neurologic/neuropathic, endocrine, and immune/autoimmune disease of widespread pain;
2. TMJ pain associated with another joint pain;
3. previous history of radiation treatment to the head and neck;
4. previous history of TMJ surgery;
5. previous history of trauma to jaw;
6. pregnancy.

**Figure 1:**
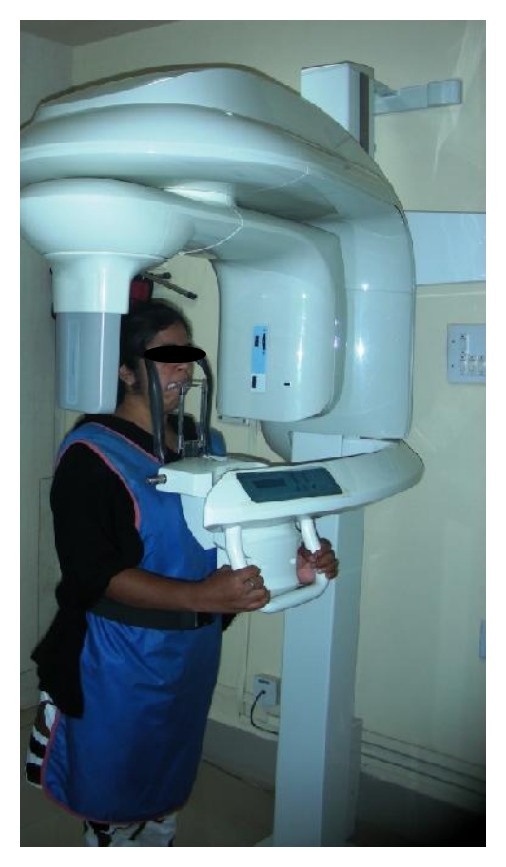
Patient positioned for DVT in Kodak 9000C 3D Extra oral imaging system.

**Figure 2:**
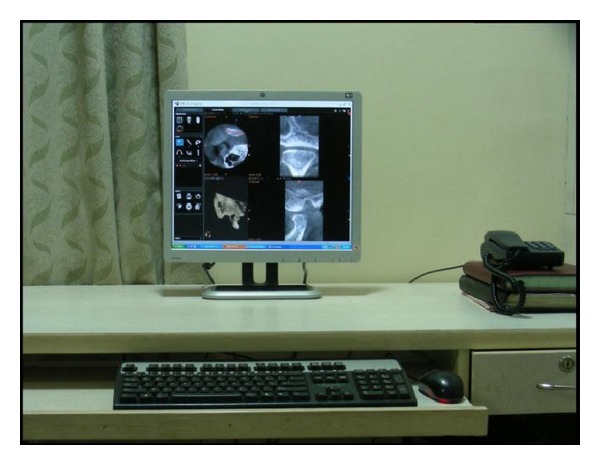
HP L1910 19-inch LCD Monitor used for interpretation of DVT and OPG images.

All the patients were subjected to conventional (OPG) and digital imaging (DVT) evaluation. KODAK 9000C 3D Extra oral imaging system (Care stream Health, Inc., 150 Verona Street, Rochester, NY 14 608) was used for obtaining both OPG and DVT images (Figures 1 and 2).

Technical specification of the machine is shown in Table 1.

### 2.1. Exposure Parameters

 (i) Panoramic radiography is as follows: 70–74 Kv, 14.3–15.1 mAs with scan time of 13.9–15.1 seconds.
 (ii) Digital volumetric tomography is as follows: 70–80 kv, 10 × 10.8 mAs with a scan time of 24 seconds.

The temporomandibular joint was assessed by both panoramic and digital volumetric tomography images. In the DVT images and 200 *μ*m tomographic sections were taken in sagittal, axial, and coronal planes. Tomographic sections were taken in the curved planar reformation (panorex), a series of multiplanar reconstructions (cross-sections) and oblique planar reformation.

### 2.2. Radiation Exposure to the Patient

Radiation exposure to the patient is as follows:

 OPG—0.01 mSv (70–85 mGy cm^2^)
 DVT—1.02 mSv to 1.05 mSv (220–235 mGy cm^2^)

The radiographic exposure for patients in both groups was well below the maximum permissible dose of 2.4 mSv as per the NCRP guidelines. Radiation safety precautions such as thyroid collar and lead apron were used before subjecting the patients for imaging evaluation.

The temporomandibular joints were assessed as follows:

 0: normal;
 1: erosion;
 2: flattening;
 3: osteophytes;
 4: sclerosis;
 5: resorption;
 6: other changes.

### 2.3. Interpretation of Radiographs

The assessment of both OPG and DVT images was done by three observers blinded to each other. OPG and DVT images were assessed separately. All three observers assessed these images twice with an interval of one week. All the three observers were asked to interpret the images to see the bony changes in the condyle and gave the code ranging from 0 to 6. All three observers interpreted the OPG and DVT images on HP L1910 19-inch square LCD Monitor with 1280 × 1024 screen resolution.

### 2.4. Statistical Analysis

SPSS 10 software is used for statistical analysis. One-way ANOVA test was done to evaluate interobserver and intraobserver variation. The two imaging modalities were compared and subjected to statistical analysis with the help of *Z* test.

## 3. Results

A total of 100 TMJ in 62 patients with a complaint of TMJ pain were assessed for the different condylar changes as mentioned in the methodology. All the 100 TMJ was assessed by two radiological methods, one being OPG and the other being DVT. Three observers assessed the diagnostic information for each of the imaging modalities. The three observers assessed the diagnostic information for each of the imaging modalities twice with an interval of one week. Distribution of gender and age in the present study is shown in Table 2. Interpretation of OPG and DVT images done by all the three observers is shown in Tables 3 and 4, respectively. There was good agreement between all the observers suggestive of no inter- and intraobserver variation (Table 5). Statistically significant difference was observed between two imaging modalities in assessing erosion and osteophytes (*P* < 0.05) which is shown in Table 6.

## 4. Discussion

Imaging is considered as an important diagnostic adjunct to the clinical assessment [10]. Several radiographic methods are used to assess bony changes that affect the TMJ. It is important to obtain a clear and precise image of the region. Superimposition of adjacent structures, different angulations of the condyle, limitation of mouth opening in some patients, presence of artifacts, and mandibular movements during the examination make the TMJ image difficult to obtain [11]. There are many radiographic techniques for TMJ examination. The most recent one is DVT or CBCT.

A recent “effective dose” survey showed that CBCT units delivered a broad range of doses (dependent on machine, field size, resolution, etc.) of between 13 Sv (minimum dose, small volume) and 82 Sv (maximum dose, large volume) which compared favorably with radiation dose inflicted by multislice CT (MSCT) of between 474 Sv and 1,160 Sv for mandibular and full head scans, respectively. To put these measurements into perspective, panoramic doses have recently been found to range between 3 and 24 Sv.

In the present study, we evaluated different bony changes of condyle seen in patient with TMJ pain using DVT and OPG. The diagnostic reliability of OPG and DVT radiographic techniques to see the condylar bony changes were assessed and compared with each other.

In our study, we used KODAK 9000C 3D Extra oral imaging system to obtain both OPG and DVT images. Using of this system can be justified by a study done by Alqerban et al. in which authors compared six different CBCT systems to assess the root resorption [12].

**Figure 3:**
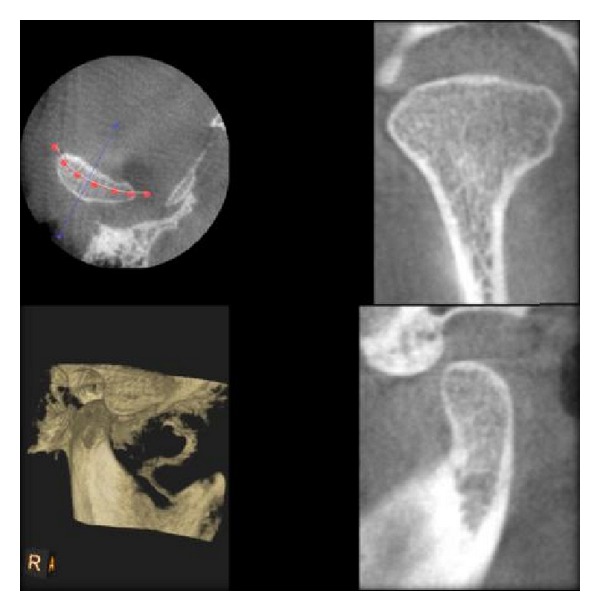
Normal condyle as seen in DVT.

**Figure 4:**
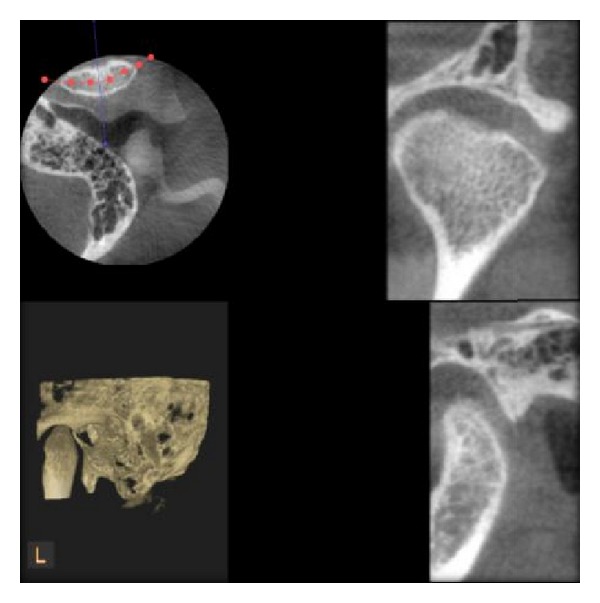
Erosion of condyle as seen in DVT.

**Figure 5:**
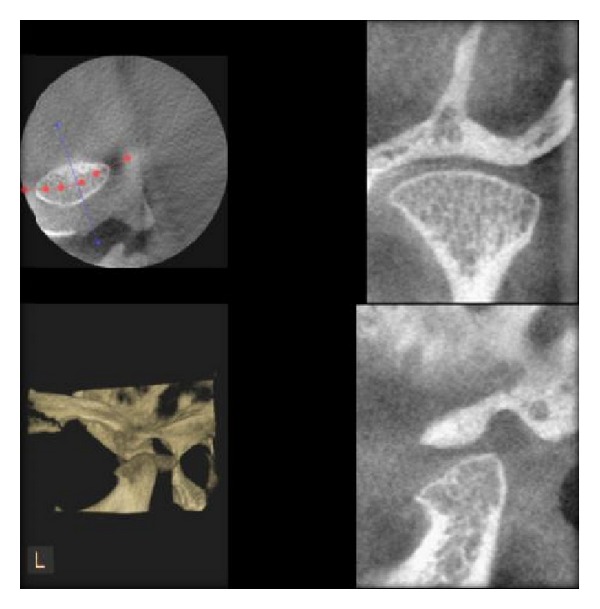
Flattening of condyle as seen in DVT.

**Figure 6:**
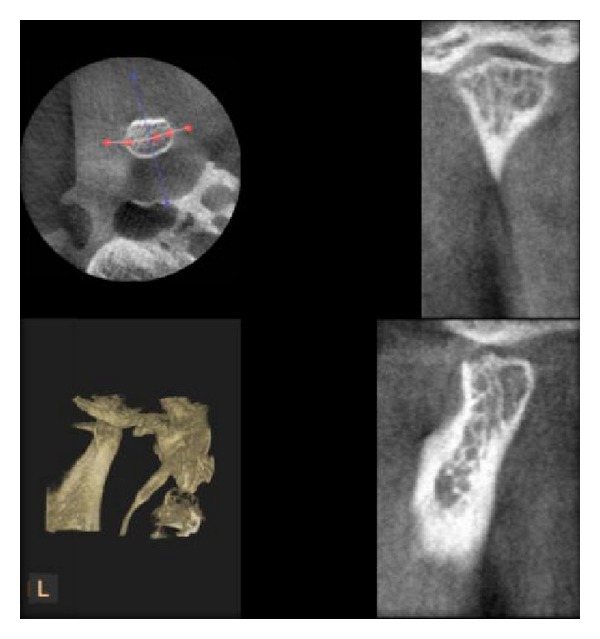
Osteophyte of condyle as seen in DVT.

**Figure 7:**
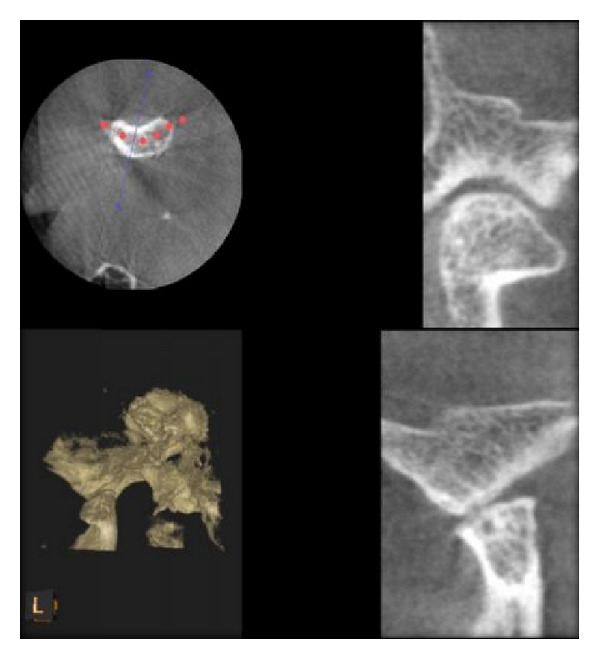
Sclerosis of condyle as seen in DVT.

**Figure 8:**
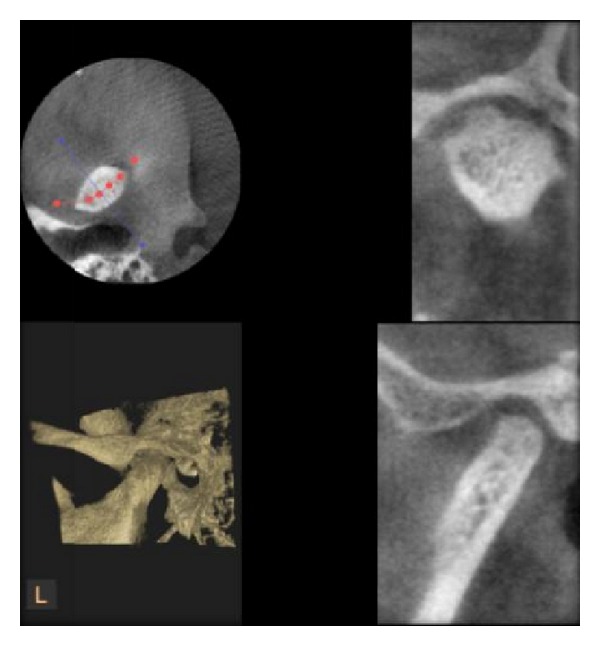
Resorption of condyle as seen in DVT.

Different bony changes assessed in our study are as follows:

- erosion;
- flattening;
- osteophytes;
- sclerosis;
- resorption;
- other changes;

Erosion is defined as an area of decreased density of the cortical bone and the adjacent subcortical bone. Flattening is defined as a flat bony contour deviating from the convex form. Osteophyte is defined as marginal bony outgrowths on the condyle. Sclerosis is defined as an area of increased density of cortical bone extending into the bone marrow. Resorption is defined as a partial loss of the condylar head [11]. All the observers interpreted the images and gave the coding from 0 to 6, where 0 stands for normal (Figure 3), 1 for erosion (Figure 4), 2 for flattening (Figure 5), 3 for osteophytes (Figure 6), 4 for sclerosis (Figure 7), 5 for resorption (Figure 8), and 6 for other changes.

In our study, we included 100 TMJ of 62 patients who had complained of TMJ pain. Out of 62 patients, 44 were female (67.75%) and 20 were male (32.25%) (Table 2). All the 100 joints were subjected for OPG and DVT examination and the images were interpreted by the three observers separately.

In our study, we had three observers to interpret the images who were blinded to each other, so that interobserver variations can be assessed. All the three observers interpreted the DVT and OPG images twice with an interval of one week to assess intraobserver's variations.

Different bony changes as seen by the three observers in DVT and OPG are shown in Tables 3 and 4.

In our study, one-way ANOVA test was done to see interobserver and intraobserver variation (Table 5). According to this test there was no interobserver and intraobserver variation in any of the imaging modality. This presumes that the observers were experienced and well calibrated in interpreting the images. Also, the image quality of OPG and DVT was par adequate not to mislead the observers.

According to available literature bony changes of TMJ are more common in female than male. In our study out of 100 joints 64% were of female and 36% were of male. Results of the present study show that more common bony changes seen like erosion, flattening, osteophytes, and sclerosis are seen more commonly in female than male (Table 7). This is in accordance with the study done by Pontual et al. (2012) [13] in which 78% were female and 22% were male. Another study done by LeResche (1997) found that pain in the temporomandibular joint is twice as common in females as in males [14]. The greater occurrence in women may be explained by the hormonal influences of estrogen and prolactin, which may exacerbate degradation of cartilage and articular bone in addition to stimulating a series of immunological responses in the TMJ [14].

In our study most common bony change seen by all the three observers was erosion followed by flattening and osteophytes. The results of our study are consistent with other studies done by Alexiou et al. (2009) [15] and Martínez Blanco et al. (2004) [16]. Objective of the present was to evaluate the efficiency of DVT in comparison with OPG in the assessment of bony condylar changes in patients of TMJ pain. Comparison of both imaging modalities was done using the *Z* test. Results of the *Z* test are shown in Table 6.

Most common bony change seen in our study is erosion. According to the results of our study there was a significant difference between DVT and OPG in assessing erosion of the condyle (*P* < 0.05). Erosion is the initial stage of degenerative changes, indicating that the TMJ is unstable and changes in bone surfaces will occur, probably resulting in changes in occlusion [11]. In our study, 61% of erosion could be identified in DVT whereas OPG was able to detect only 21%. Hence DVT has proved to be more superior than OPG in detecting early bony changes like erosion. This can be explained by the fact that DVT is a three-dimensional imaging technique in which the images can be viewed in all the three sections, that is, axial, coronal, and sagittal. The thickness section is 200 *μ*m. Hence minor changes can be well appreciated by this technique. Our results are in accordance with a study done by Lee et al. who compared the panoramic radiograph and cone beam computed tomographic images in detecting bony changes in patients with temporomandibular joint disorder and they found that CBCT was able to detect more percentage of erosion compared with OPG [17].

Second most common change observed in our study is flattening. Flattening is considered a degenerative alteration resulting from overload on the TMJ and it may be related to the involvement of the masseter and temporal muscles [11]. In our study, though DVT could detect more number of flattening than OPG, there was no statistically significant difference between two modalities in detecting this bony change. This can be explained by the fact that flattening is a gross change which can be easily detected by two-dimensional imaging like OPG. In a study done by Lee et al., they also found that OPG was able to detect more percentage of flattening [17].

Next common bony change detected in the present study was the presence of osteophytes.

Osteophytes occur in an advanced stage of degenerative change when the body adapts itself to repair the joint. The osteophytes appear to stabilize and widen the surface in an attempt to improve the overload resulting from occlusal forces, representing areas of neoformed cartilage [11]. There was statistically significant difference between DVT and OPG in detecting osteophytes. These results are similar to a study by Lee et al. in which out of 212 joints CBCT was able to detect 2.1% of osteophytes whereas OPG detected only 0.9% and hence proving that CBCT is superior to OPG [17].

Sclerosis and resorption are the other two bony changes seen in our study. DVT was able to detect more number of sclerosis and resorption compared to OPG. But there was no statistically significant results between the two imaging modality. Reason for no significant difference can be attributed to the small percentage of these changes observed in our study.

**Figure 9:**
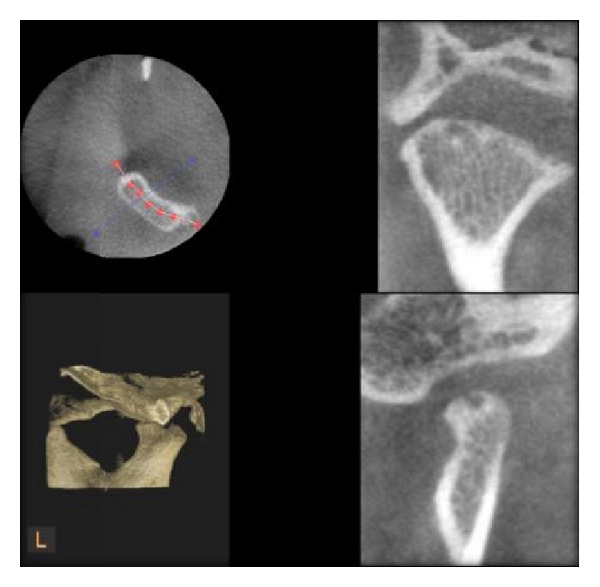
Ely's cyst of condyle as seen in DVT.

**Figure 10:**
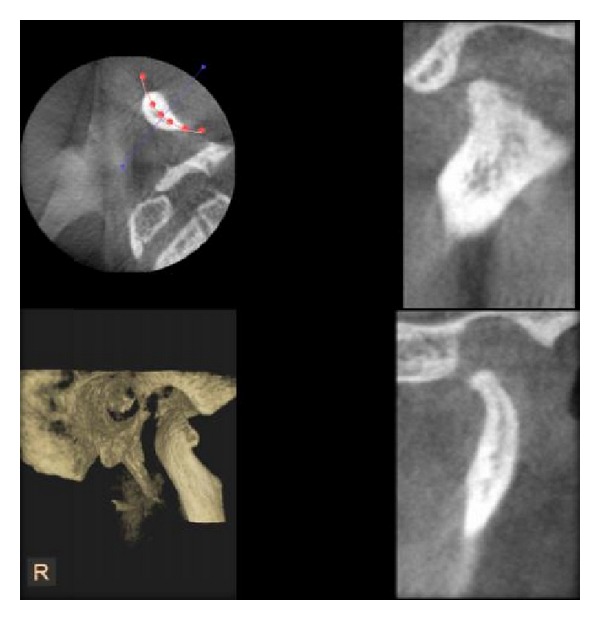
Pointed shape of condyle as seen in DVT.

**Figure 11:**
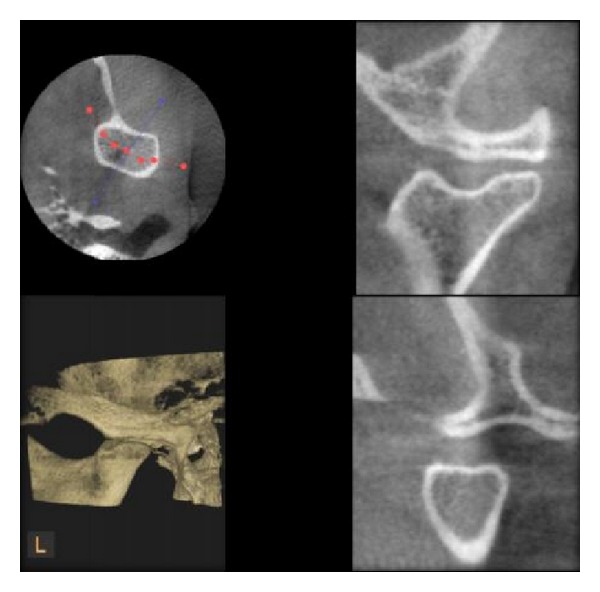
Bifid condyle as seen in DVT.

In our study, we also found other changes like Ely's cyst of the mandibular condyle (Figure 9), pointed shape of condylar head (Figure 10) and bifid condyle (Figure 11). Ely's cysts are also called subcortical cysts. These are rounded radiolucent areas that may be just below the cortical plate or deep in trabecular bone. There was no significant difference between DVT and OPG to detect Ely's cysts. Out of 100 joints we found 2 joints having Ely's cysts. In a cross-sectional study done by Mathew et al. to see condylar changes and its association with age, TMD, and dentition status, they found 5 Ely's cysts out of 75 subjects [18].

In our study there were 2 joints with a pointed condylar head which was seen in both DVT and OPG. In a study done by Christiano Oliveira et al. 22.9% (129 condyles out of 566 condyles) of condyle had pointed condylar head [19]. A smaller amount of occurrence of pointed condylar head in our study may be due to a smaller number of sample sizes.

Bifid condyle is a rare anatomic variation of mandibular condyle. It can be symptomatic or diagnosed incidentally on routine radiographic examination. They appear to be more common on the left side in unilateral cases [20]. In a study done by Cağlayan and Tozoğlu in the year 2012 they found 2 bifid condyles out of 45 subjects [21]. In our study, we found one bifid mandibular condyle in left TMJ which was detected on DVT and not in OPG. This difference may have been due to the superiority of DVT for analyzing the TMJ region because of the absence of superimposition of anatomical structures.

To conclude, OPG alone can be used to detect gross bony changes of condyle like flattening and pointed condyle and DVT is helpful in detecting changes of condyle like erosion and osteophytes and Common bony changes seen in our study are erosion followed by flattening and osteophytes both in OPG and DVT. All the bony changes are seen more commonly in female than male. Other bony changes observed in our study are Ely's cyst, pointed condyle, and bifid condyle.

## Figures and Tables

**Table 1:** 

X-ray generator	
Tube voltage	60–90 kv (max), pulsed mode for 3D modality
Tube current	2–15 mA (max)
Frequency	140 kHz (max)
Tube focal spot	0.5 mm
Total filtration	>2.5 mm eq.Al

Panoramic modality	

Technology	Digital volumetric tomography (DVT)
Sensor technology	(i) CCD
	(ii) Optical fibre sensor with Csi coating
Sensor matrix	61 × 1244 pixels
Image field	6.3 × 129.4 mm
Gray scale	16384—14 bits
Magnification	1.27

3D modality	

Technology	Digital volumetric tomography (DVT)
Sensor technology	(i) CMOS
	(ii) Optical fibre sensor with Csi coating
Gray scale	16384—14 bits
Volume size	50 × 37 mm
Voxel size	76.5 × 76.5 × 76.5 *µ*m

**Table 2:** Distribution of gender and age in this study.

Gender	Number	Age (average)	Range
Male	20	25.8 years	(15–49)
Female	42	30.5 years	(16–72)

Total	62	28.15 years	(15–72)

**Table 3:** Bony changes seen by three observers in OPG.

Condylar changes	Observer 1		Observer 2		Observer 3	
	Day 1	Day 7	Day 1	Day 7	Day 1	Day 7
Normal	56	55	55	54	58	59
Erosion	21	20	21	21	21	20
Flattening	29	29	30	28	26	25
Osteophytes	7	7	6	6	7	7
Sclerosis	5	5	5	5	6	6
Resorption	1	1	1	1	1	1
Others changes	4	4	4	4	4	4

**Table 4:** Bony changes seen by three observers in DVT.

Condylar changes	Observer 1		Observer 2		Observer 3	
	Day 1	Day 7	Day 1	Day 7	Day 1	Day 7
Normal	56	55	55	54	58	59
Erosion	21	20	21	21	21	20
Flattening	29	29	30	28	26	25
Osteophytes	7	7	6	6	7	7
Sclerosis	5	5	5	5	6	6
Resorption	1	1	1	1	1	1
Others changes	4	4	4	4	4	4

**Table 5:** One-way ANOVA test to see interobserver variation in DVT and OPG.

	*N*	Mean	Std. deviation	Std. error	*F*	*P* value
DVT						
Observer 1	100	1.4900	0.797	7.977*E* − 02	0.102	0.903
Observer 2	100	1.5400	0.845	8.459*E* − 02		
Observer 3	100	1.5300	0.846	8.463*E* − 02		
Total	**300**	**1.5200**	**0.827**	4.779**E** − 02		

OPG						
Observer 1	100	1.1300	0.393	3.933*E* − 02	0.22	0.978
Observer 2	100	1.1300	0.393	3.933*E* − 02		
Observer 3	100	1.1200	0.383	3.835*E* − 02		
Total	**300**	**1.1267**	**0.388**	2.244**E** − 02		

**Table 6:** Comparison of DVT and OPG groups.

*Z* Test			
Comparison of DVT and OPG groups			*P* value
Condylar changes	OPG	DVT	
Normal	56	22	0.000∗
Erosion	21	61	0.000∗
Flattening	29	35	0.449
Osteophytes	4	20	0.001∗
Sclerosis	5	10	0.283
Resorption	1	4	0.369
Others changes	3	5	1.000

*Statistically significant difference was observed between two imaging modality in assessing erosion and osteophytes as *P* value is less than 0.05.

**Table 7:** Most common bony changes in male and female in OPG and DVT.

Condylar changes	OPG			DVT		
	Male	Female	Total	Male	Female	Total
Erosion	8	13	21	21	40	61
Flattening	11	18	29	15	20	35
Osteophytes	3	4	7	9	11	20
Sclerosis	2	3	5	3	7	10
